# Factors associated with testing for HIV and other sexually transmitted infections in men who have sex with men and transgender women in Bangkok, Thailand

**DOI:** 10.1186/s12981-022-00449-0

**Published:** 2022-06-21

**Authors:** Trevor A. Crowell, Sorachai Nitayaphan, Narongrid Sirisopana, Tanyaporn Wansom, Suchai Kitsiripornchai, Leilani Francisco, Qun Li, Nicole Dear, Robert J. O’Connell, Punnee Pitisuttithum, Sandhya Vasan, for the RV348B Study Group

**Affiliations:** 1 U.S. Military HIV Research Program, Walter Reed Army Institute of Research, Silver Spring, MD USA; 2 Henry M. Jackson Foundation for the Advancement of Military Medicine, Bethesda, MD USA; 3 Armed Forces Research Institute of Medical Sciences, Bangkok, Thailand; 4 Mahidol University, Bangkok, Thailand; 5 Present Address: Dreamlopments Social Enterprise and Foundation, Bangkok, Thailand

**Keywords:** Screening practices, Sexual and gender minorities, Human immunodeficiency virus, Testing practices, Screening practices, Voluntary counseling and testing, Early diagnosis, Healthcare acceptability

## Abstract

**Background:**

Routine screening for HIV and other sexually transmitted infections (STIs) facilitates early diagnosis and treatment, thereby preventing morbidity and onward transmission. We estimated the prevalence of prior HIV/STI testing among men who have sex with men (MSM) and transgender women (TGW) in Bangkok, Thailand, and identified factors associated with prior testing.

**Methods:**

Cross-sectional analyses were performed using data collected at enrollment into an HIV incidence cohort. From April to October 2017, MSM and TGW were enrolled if they were aged 18–35 years, reported anal intercourse with a male or TGW partner, and reported behavioral vulnerability to HIV. Participants answered questions about demographics, sexual behaviors, and lifetime HIV/STI testing history. Multivariable robust Poisson regression was used to estimate risk ratios (RRs) and 95% confidence intervals (CIs) for factors potentially associated with prior testing.

**Results:**

Among 1,014 participants, 348 (34.3%) were TGW and the median age was 21.6 (interquartile range 20.0-24.8) years. Prior testing for HIV was reported by 421 (41.5%) and for other STIs by 268 (26.4%). HIV testing was more common among participants aged ≥ 22 years (RR 1.37 [95% CI 1.13–1.67]), with college education as compared to secondary or less (RR 1.37 [95% CI 1.08–1.72]), and who met male sexual partners online (RR 1.52 [95% CI 1.24–1.85]), but lower among participants attracted to both men and women as compared to men only (RR 0.64 [95% CI 0.51–0.81]) and who met male sexual partners in bars (RR 0.83 [95% CI 0.72–0.97]). Similar associations were observed with prior testing for other STIs, including increased testing among participants with college education (RR 1.52 [95% CI 1.11–2.09]) and who met male sexual partners online (RR 1.73 [95% CI 1.30–2.31]), but lower among participants attracted to both men and women (RR 0.70 [95% CI 0.51–0.96]) and who met male sexual partners in bars (RR 0.67 [95% CI 0.54–0.83]).

**Conclusions:**

Despite behavioral vulnerability, prior testing for HIV and other STIs was uncommon. Online engagement strategies may be effectively reaching Thai MSM and TGW who meet sexual partners online, but new interventions are needed to encourage testing among younger, less educated, and bisexual MSM and TGW.

## Introduction

The Joint United Nations Programme on HIV/AIDS (UNAIDS) has set a target for zero new HIV infections by 2030 as part of its Sustainable Development Goals. Routine testing of vulnerable individuals is crucial to curbing the spread of human immunodeficiency virus (HIV) and other sexually transmitted infections (STIs). Early diagnosis of persons living with HIV (PLWH) facilitates rapid initiation of antiretroviral therapy to reduce HIV-related morbidity [1–3] and prevent onward HIV transmission [4–6]. HIV transmission can also be reduced by the early diagnosis and treatment of concurrent bacterial STIs [7, 8]. Amongst people with and without HIV, detecting and treating bacterial STIs can prevent the development of long-term sequelae such as pelvic inflammatory disease, proctitis, and their potentially devastating complications [9, 10]. Testing services for HIV and other STIs can be co-located to efficiently reach vulnerable populations [11–13] and can be leveraged to link vulnerable individuals to biologic and behavioral prevention programs [14–16].

Access to routine testing is particularly important for sexual and gender minority populations such as men who have sex with men (MSM) and transgender women (TGW), who are disproportionately affected by HIV and other STIs [17, 18]. These two groups are often conflated [19, 20], but it is increasingly recognized that individuals who were assigned male sex at birth and identify as men (cisgender men) exhibit different sexual behaviors and other characteristics from individuals who were assigned male sex at birth and identify as women (transgender women), even when both engage in sex with men [21, 22]. Guidelines in the United States and Europe recommend annual testing of sexually active cisgender men and TGW who have sex with men for HIV and other STIs with consideration for more frequent testing [23, 24]. Additionally, in the United States, testing every three months is recommended for those who are prescribed HIV pre-exposure prophylaxis (PrEP) [25] and in Thailand such testing is recommended every six months [26]. Despite such recommendations, HIV diagnosis is often delayed until relatively late in the disease course [27–32] and many asymptomatic bacterial STIs go undiagnosed in routine care [32–36], particularly in resource-limited settings. Prior evidence from settings other than Thailand suggests that HIV testing is more common among people with higher education, HIV-related knowledge, and self-recognition of HIV risk [37–40].

Thailand has been a global leader in research on the prevention and treatment of HIV [41–44] and was one of the first Southeast Asian countries to recommend PrEP for MSM who are behaviorally vulnerable to HIV [45]. Despite this, PrEP uptake has been low [46, 47]. While HIV incidence among MSM in Bangkok appears to be declining, data suggest increasing HIV diagnoses among TGW since 2014 [48]. MSM and TGW in Bangkok also have a high burden of predominantly asymptomatic chlamydia and gonorrhea infections that would go undetected without routine testing [49, 50]. We estimated the prevalence of self-reported prior testing for HIV and other STIs among MSM and TGW in Bangkok, Thailand. We identified factors associated with prior testing for HIV and other STIs in order to inform the design of targeted interventions to increase testing uptake.

## Methods

### Study population

Cross-sectional analyses were performed using data collected at enrollment into an HIV incidence cohort. From April through October 2017, participants were enrolled at two sites in Bangkok, Thailand, as previously described [51]. Bangkok is a city with highly visible sexual and gender minority populations who, despite an outward appearance of acceptance by the community, still face stigma and discrimination that create barriers to healthcare engagement [52]. Recruitment activities at the Royal Thai Army Clinical Research Centre site included social media advertisements for free HIV testing and direct outreach to bars, saunas, and other entertainment venues. Recruitment activities at the Vaccine Trial Centre at Mahidol University included community engagement via health campaigns and festivals as well as referrals from community-based organizations. Both sites conducted recruitment activities at universities and encouraged peer referrals. Eligible participants were cisgender men or TGW, aged 18–35 years, who reported anal intercourse with a male or TGW partner and one or more of the following criteria in the six months prior to enrollment: (1) engaging in condomless anal intercourse with a male or TGW partner living with HIV or with unknown HIV status; (2) having three or more sexual partners; (3) exchanging sex for money, goods, or drugs; or (4) being diagnosed with a new STI such as syphilis, gonorrhea, chlamydia, or herpes. These criteria were selected based on prior studies showing higher HIV and STI incidence in people from this age group and with these sexual behaviors [53–55]. Participants were excluded if they were living with HIV or had previously received an investigational HIV vaccine candidate. Participants received risk reduction counseling and underwent testing for HIV and syphilis at three, six, 12, and 18 months after enrollment. Participants who were diagnosed with HIV were referred to a hospital clinic accessible via the national health care benefit; study staff provided counseling to facilitate engagement in care and antiretroviral treatment initiation.

All participants provided written informed consent in either Thai or English. The study was approved by institutional review boards at Walter Reed Army Institute of Research, Silver Spring, MD, USA; Faculty of Tropical Medicine at Mahidol University, Bangkok, Thailand; and the Royal Thai Army, Bangkok, Thailand.

### Data collection

Data for these cross-sectional analyses were collected at the screening visit for the prospective HIV incidence cohort study, which included a computer-assisted self-interview (CASI) questionnaire that collected detailed information regarding participant demographics, sexual behaviors, and STI history. The CASI approach reduced the potential for errors in transcribing participant responses, ensured consistent administration of the questionnaire, and allowed for programming of skip patterns and logic checks to optimize data validity. Data collected on case report forms were compiled into a password-protected electronic database and underwent 100% verification against source documents for key variables (demographics, eligibility, HIV test results, syphilis test results, and end of study disposition) by a trained clinical study monitor.

The outcome variables of interest for these analyses were (1) prior lifetime history of HIV testing and (2) prior lifetime history of testing for other STIs. Prior testing history was assessed by the questions “Have you ever been tested for HIV?” and “Have you ever been tested for sexually transmitted infections, or STIs? (STIs can include syphilis, gonorrhea, chlamydia, herpes.)” If yes, participants were asked whether they had ever been diagnosed with an STI by a doctor or clinic. If so, they were asked whether they had specifically ever been diagnosed with syphilis, gonorrhea, chlamydia, and/or herpes and when they had most recently received an STI diagnosis with answer choices of within three months, within six months, within one year, or more than one year prior.

Independent variables evaluated in these analyses included age, gender identity, sexual attraction, education level, income, and venues for meeting male sexual partners (bars, saunas, online). These variables were assessed by self-report via the CASI questionnaire. Gender was categorized using a validated two-step method that included asking participants which sex they were assigned at birth (male or female) and how they described their current gender identity (male, female, transgender woman, or transgender man) [56, 57]. If a participant responded with both sex assigned at birth and current identity as male, the participant was characterized as a cisgender MSM. If a participant responded with sex assigned at birth as male and gender identity as either female or TGW, the participant was categorized as a TGW. For these cross-sectional analyses of data collected at the screening visit for a longitudinal study, fluidity of gender during longitudinal follow-up was not considered.

### Statistical analyses

All enrolled participants who answered both questions about prior testing were included in these analyses. Comparisons between groups of interest were made using the Chi-squared test for categorical variables or Student’s t-test for continuous variables. In separate analyses for prior HIV testing and prior STI testing, unadjusted and adjusted robust Poisson regression models were used to estimate risk ratios (RRs) and 95% confidence intervals (CIs) for pre-specified factors potentially associated with any lifetime history of testing [58]. All pre-specified factors were included in the adjusted multivariable models, regardless of significance in unadjusted modeling. Analyses were performed using Stata 15.1 (StataCorp LP, College Station, TX).

## Results

### Characteristics of the study population

A total of 1,014 enrolled participants answered both questions about prior testing and were included in these analyses. These participants had a median age of 21.6 (interquartile range [IQR] 20.0–24.8) years. The study population included 657 (64.8%) cisgender men, 348 (34.3%) TGW, and 9 (0.9%) participants with other/unknown gender identity. Sexual attraction mostly or only to men was reported by 790 (77.9%) participants, both men and women by 197 (19.4%), and women only by 27 (2.7%). Participants reported a median of 5 (IQR 3–8) male or TGW sexual partners in the six months prior to enrollment. Any lifetime history of prior testing for HIV was reported by 421 (41.5%) and for other STIs by 268 (26.4%; Table 1).

**Table 1:** Study population characteristics, overall and by lifetime history of testing for hiv or other sexually transmitted infections

	Overall	Any HIV Testing		P	Any Other STI Testing		**P**
	(n = 1014)	No (n = 593)	Yes (n = 421)		No (n = 746)	Yes (n = 268)	
*Age*				**< 0.001**			**< 0.001**
< 22 years	541 (53.4%)	357 (60.2%)	184 (43.7%)		426 (57.1%)	115 (42.9%)	
≥ 22 years	473 (46.6%)	236 (39.8%)	237 (56.3%)		320 (42.9%)	153 (57.1%)	
*Gender Identity*				0.18			0.12
Cisgender Man	657 (64.8%)	378 (63.7%)	279 (66.3%)		482 (64.6%)	175 (65.3%)	
Transgender Woman	348 (34.3%)	212 (35.8%)	136 (32.3%)		260 (34.9%)	88 (32.8%)	
Missing/Unknown/Other	9 (0.9%)	3 (0.5%)	6 (1.4%)		4 (0.5%)	5 (1.9%)	
*Sexual Attraction*				**< 0.001**			**0.004**
Men Only	790 (77.9%)	431 (72.7%)	359 (85.3%)		562 (75.3%)	228 (85.1%)	
Both Men and Women	197 (19.4%)	142 (23.9%)	55 (13.1%)		161 (21.6%)	36 (13.4%)	
Women Only	27 (2.7%)	20 (3.4%)	7 (1.7%)		23 (3.1%)	4 (1.5%)	
*Highest Education Level*				**< 0.001**			**< 0.001**
Secondary or Less	202 (19.9%)	135 (22.8%)	67 (15.9%)		158 (21.2%)	44 (16.4%)	
Vocational School	65 (6.4%)	43 (7.3%)	22 (5.2%)		54 (7.2%)	11 (4.1%)	
Some University	540 (53.3%)	330 (55.6%)	210 (49.9%)		414 (55.5%)	126 (47.0%)	
Bachelor’s Degree or Higher	207 (20.4%)	85 (14.3%)	122 (29.0%)		120 (16.1%)	87 (32.5%)	
*Income*				**< 0.001**			**< 0.001**
< 15,000 THB per month	662 (65.3%)	414 (69.8%)	248 (58.9%)		511 (68.5%)	151 (56.3%)	
≥ 15,000 THB per month	349 (34.4%)	176 (29.7%)	173 (41.1%)		232 (31.1%)	117 (43.7%)	
Missing/Unknown	3 (0.3%)	3 (0.5%)	0 (0%)		3 (0.4%)	0 (0%)	
*Meets Male Sexual Partners at Bars*				**0.011**			**< 0.001**
No	581 (57.3%)	320 (54.0%)	261 (62.0%)		403 (54.0%)	178 (66.4%)	
Yes	433 (42.7%)	273 (46.0%)	160 (38.0%)		343 (46.0%)	90 (33.6%)	
*Meets Male Sexual Partners at Saunas*				**0.014**			**0.028**
No	929 (91.6%)	554 (93.4%)	375 (89.1%)		692 (92.8%)	237 (88.4%)	
Yes	85 (8.4%)	39 (6.6%)	46 (10.9%)		54 (7.2%)	31 (11.6%)	
*Meets Male Sexual Partners Online*				**< 0.001**			**< 0.001**
No	283 (27.9%)	203 (34.2%)	80 (19.0%)		237 (31.8%)	46 (17.2%)	
Yes	731 (72.1%)	390 (65.8%)	341 (81.0%)		509 (68.2%)	222 (82.8%)	

Participants in Bangkok, Thailand, were categorized based on self-report of any lifetime history of testing for HIV and for other sexually transmitted infections. Data are presented as n (column %). Comparisons between groups with no history of testing and those with any history of testing were made using the Chi-squared test. Statistically significant p-values (p < 0.05) are shown in bold

### Prior STI diagnoses

**Fig. 1:**
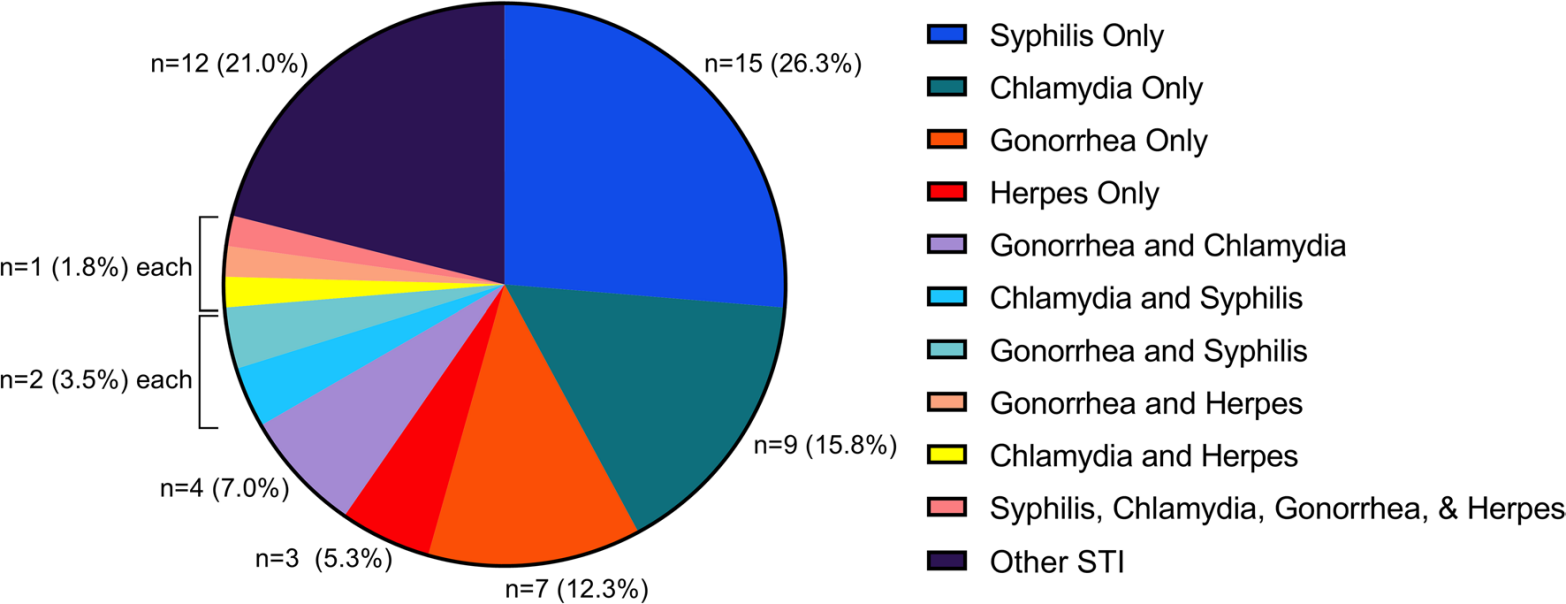
Of 268 participants who reported previously being tested for a sexually transmitted infection, 57 (21.3%) reported that they had been diagnosed with a sexually transmitted infection by a doctor or clinic. These participants were asked to specify whether they had been diagnosed with any or all of the following: syphilis, gonorrhea, chlamydia, and/or herpes. Twelve participants reported a history of sexually transmitted infection but did not indicate any of the solicited diagnoses

Among the 268 participants who reported any lifetime history of testing for STIs other than HIV, a prior positive test result was self-reported by 57 (21.3%). Specifically, a prior diagnosis of syphilis was reported by 20 previously-tested participants (7.5%), chlamydia by 17 (6.3%), gonorrhea 15 (5.6%), and herpes by 6 (2.2%). Among the 57 participants with a prior STI diagnosis, 12 (21.0%) reported a prior positive STI test result but none of the specific STIs solicited, 34 (59.6%) reported just one of the specific STI diagnoses, 10 (17.5%) reported two different STI diagnoses, and one (1.8%) reported a history of all four solicited STIs (Fig. 1). Among these 57 participants, 12 (21.0%) stated that their most recent STI was within the preceding three months, 13 (22.8%) in the preceding 3–6 months, 14 (24.6%) in the preceding 6–12 months, and 18 (31.6%) more than one year prior to enrollment.

### Factors associated with any lifetime history of HIV or STI Testing

After adjusting for potentially confounding factors, HIV testing uptake was higher among participants aged ≥ 22 years as compared to < 22 years (RR 1.37 [95% CI 1.13–1.67]), with college education as compared to secondary or less (RR 1.37 [95% CI 1.08–1.72]), and who met male sexual partners online (RR 1.52 [95% CI 1.24–1.85]), but lower among participants attracted to both men and women as compared to men only (RR 0.64 [95% CI 0.51–0.81]) and who met male sexual partners in bars (RR 0.83 [95% CI 0.72–0.97]; Table 2). Gender identity, income, and meeting male sexual partners at saunas were not associated with prior testing for HIV in the multivariable model. Similar associations were observed with prior testing for other STIs, including increased testing among participants with college education (RR 1.52 [95% CI 1.11–2.09]) and who met male sexual partners online (RR 1.73 [95% CI 1.30–2.31]), but lower testing among participants attracted to both men and women (RR 0.70 [95% CI 0.51–0.96]) and who met male sexual partners in bars (RR 0.67 [95% CI 0.54–0.83]). Age, gender identity, income, and meeting male sexual partners at saunas were not associated with prior testing for other STIs.

**Table 2:** Factors associated with testing for HIV and other sexually transmitted infections testing among cisgender men and transgender women who have sex with men in Bangkok, Thailand (n = 1014)

	Any HIV Testing		Any Other STI Testing	
	Unadjusted Risk Ratio (95% Confidence Interval)	Adjusted Risk Ratio (95% Confidence Interval)	Unadjusted Risk Ratio (95% Confidence Interval)	Adjusted Risk Ratio (95% Confidence Interval)
*Age*				
< 22 years	Reference	-	Reference	**-**
≥ 22 years	**1.47 (1.27–1.71)**	**1.37 (1.13–1.67)**	**1.52 (1.24–1.87)**	1.21 (0.90–1.61)
*Gender Identity*				
Cisgender Man*	Reference	-	Reference	**-**
Transgender Woman	0.91 (0.78–1.07)	0.87 (0.74–1.02)	0.94 (0.75–1.16)	0.93 (0.74–1.17)
*Sexual Attraction*				
Men Only	Reference	-	Reference	**-**
Both Men and Women	**0.61 (0.48–0.78)**	**0.64 (0.51–0.81)**	**0.63 (0.46–0.87)**	**0.70 (0.51–0.96)**
Women Only	0.57 (0.30–1.08)	0.60 (0.33–1.09)	0.51 (0.21–1.28)	0.58 (0.25–1.34)
*Highest Education Level*				
Secondary or Less	Reference	-	Reference	**-**
Vocational School	1.02 (0.69–1.51)	1.05 (0.73–1.52)	0.78 (0.43–1.41)	0.82 (0.46–1.46)
Some University	1.17 (0.94–1.46)	1.26 (0.99–1.60)	1.07 (0.79–1.45)	1.12 (0.80–1.57)
Bachelor’s Degree or Higher	**1.78 (1.42–2.23)**	**1.37 (1.08–1.72)**	**1.93 (1.42–2.62)**	**1.52 (1.11–2.09)**
*Income*				
< 15,000 THB per month*	Reference	-	Reference	**-**
≥ 15,000 THB per month	**1.33 (1.15–1.54)**	1.16 (0.98–1.36)	**1.48 (1.20–1.81)**	1.24 (0.98–1.57)
*Meets Male Sexual Partners at Bars*				
No	Reference	-	Reference	**-**
Yes	**0.82 (0.71–0.96)**	**0.83 (0.72–0.97)**	**0.68 (0.54–0.85)**	**0.67 (0.54–0.83)**
*Meets Male Sexual Partners at Saunas*				
No	Reference	-	Reference	**-**
Yes	**1.34 (1.08–1.66)**	1.14 (0.92–1.41)	**1.43 (1.06–1.93)**	1.23 (0.90–1.66)
*Meets Male Sexual Partners Online*				
No	Reference	-	Reference	**-**
Yes	**1.65 (1.35–2.02)**	**1.52 (1.24–1.85)**	**1.87 (1.40–2.49)**	**1.73 (1.30–2.31)**

* participants with missing/unknown data (< 0.5%) were included in the reference category

Unadjusted and adjusted Poisson regression with robust error variance was used to estimate risk ratios and 95% confidence intervals for pre-specified factors potentially associated with prior testing. All listed variables were included in the adjusted multivariable models for lifetime history of HIV testing and lifetime history of other STI testing prior to study enrollment. Statistically significant (p < 0.05) risk ratios are shown in bold

## Discussion

Despite recruitment based on behavioral vulnerability to HIV, prior testing for HIV was observed in fewer than half of participants in our study. This is similar to what has been reported in several prior studies of Thai MSM and TGW [59–61]. Prior testing for other STIs was even less common in our study, despite a high number of cases detected among the minority of participants who had been previously tested. MSM and TGW in Bangkok have experienced recent surges or outbreaks of STIs such as syphilis [62] and hepatitis C [63]. Early diagnosis and treatment of such infections is critical to interrupting chains of transmission.

We found that participants who met their male sexual partners online were more likely to have been tested for both HIV and other STIs. One prior study that recruited MSM in Bangkok who used mobile applications to find sexual partners found higher testing uptake than was observed in our overall study population, with about two-thirds reporting prior testing for HIV and three-quarters reporting prior testing for other STIs [64]. In Thailand and much of Southeast Asia, smartphones and high-speed internet are frequently used to socialize and find sexual partners [64–66]. These digital platforms can be leveraged to connect vulnerable populations to HIV and STI testing services. For example, online sex-seeking platforms could be used for targeted marketing and promotions to incentivize HIV and STI testing [67]. Motivational interviewing is a goal-oriented counseling technique that can potentially be delivered over the phone [68] and has been used to successfully promote HIV testing among MSM in Hong Kong [69]. HIV self-testing with online supervision may be appropriate for young MSM who seek sexual partners online but are disinterested in venue-based testing [70]. Many of these types of interventions are already being deployed in Bangkok and their success probably contributes to the increased testing uptake observed amongst participants in our study who met sexual partners online.

Conversely, we identified gaps in testing uptake among participants who met sexual partners outside of online settings, particularly those who met male sexual partners at bars. Decentralization of HIV and STI testing services may increase accessibility of these services to people who are not currently seeking testing in traditional healthcare settings or being reached by targeted online interventions. For example, lay providers can be successfully trained to provide rapid diagnostic testing for HIV [71] and community-based organizations can be leveraged to deliver HIV and STI prevention packages to venues frequented by behaviorally vulnerable MSM and TGW [47]. When designing public health interventions to increase HIV and STI testing, providing a combination of options that range from predominantly online engagement to predominantly in-person may best address the needs and preferences of diverse populations in need of testing [72]. Strategies for successful linkage to care after HIV or STI diagnosis outside of traditional healthcare settings will need to be carefully considered [73].

We found that prior testing for HIV and other STIs was most common among participants who reported sexual attraction exclusively to men, revealing a potential gap in accessibility of testing to individuals with other sexual preferences. It is important to note that all participants in this study, regardless of sexual attraction, reported sex with male or TGW partners. Bisexual or heterosexual individuals who engage in same-sex sexual practices may feel particularly marginalized or stigmatized and be less likely to access MSM-focused venues for HIV or STI testing [74–76]. Healthcare providers and organizations must recognize the diversity of sexual preferences represented amongst MSM and TGW populations in order to create welcoming spaces and differentiated methods for delivering testing services to sexual and gender minority populations that may otherwise be overlooked.

Knowledge about HIV and other STIs influences risk perception and subsequent testing uptake [77]. This may at least partly explain the relationship between higher education and increased testing in our study. Prior studies have shown that discordance between self-perceived and actual risk may be a barrier to uptake of testing services by Thai MSM and TGW [78] as well as MSM travelers to Thailand [79]. In one study of MSM attending saunas in Bangkok, younger participants were more likely than older ones to demonstrate sexual behaviors associated with HIV risk but had a false perception of low HIV risk [80]. Interventions to increase knowledge about HIV and other STIs, delivered in early academic settings and outside of academic settings, may help to improve testing uptake.

It should be noted that most of the data for these analyses were assessed by self-report and could be susceptible to both recall and social desirability biases; use of CASI for data collection should have reduced the latter. Data were collected cross-sectionally at enrollment into a study that included routine HIV and syphilis screening, so analyses were based primarily on historic experiences and temporal associations could not be evaluated. The study was conducted at two sites in Bangkok with experience engaging sexual and gender minority populations, so findings may not be generalizable to other settings. Recruitment activities that focused on bars, saunas, and online networking platforms may have enriched the study population for participants who met sexual partners via these venues.

## Conclusions

We observed substantial room for improvement in testing uptake for HIV and other STIs among MSM and TGW in Bangkok, Thailand. Differentiated models of service delivery that leverage online and offline opportunities for engagement will be needed to reach diverse, marginalized populations that are behaviorally vulnerable to HIV and other STIs.

## Data Availability

The Henry M. Jackson Foundation for the Advancement of Military Medicine (HJF) and the Water Reed Army Institute of Research (WRAIR) are committed to safeguarding the privacy of research participants. Distribution of data will require compliance with all applicable regulatory and ethical processes, including establishment and approval of an appropriate data-sharing agreement. To request a minimal data set, please contact the data coordinating and analysis center at PubRequest@hivresearch.org and indicate the RV348B study along with the name of the manuscript.

## Abbreviations

AIDS: Acquired immune deficiency syndrome
AR: Army Regulation
CASI: Computer-assisted self-interview
CFR: Code of Federal Regulations
CI: Confidence intervals
HIV: Human immunodeficiency virus
MSM: Men who have sex with men
PrEP: Pre-exposure prophylaxis
RR: Risk ratio
STIs: Sexually transmitted infections
TGW: Transgender women
UNAIDS: Joint United Nations Programme on HIV/AIDS
U.S.: United States
